# Use of artificial intelligence to enable dark nudges by transnational food and beverage companies: analysis of company documents

**DOI:** 10.1017/S1368980022000490

**Published:** 2022-05

**Authors:** Ruby Brooks, Duy Nguyen, Asim Bhatti, Steven Allender, Michael Johnstone, Chee Peng Lim, Kathryn Backholer

**Affiliations:** 1 Global Obesity Centre, Institute for Health Transformation, School of Health and Social Development, Deakin University, Geelong, VIC 3220, Australia; 2 Institute for Intelligent Systems Research and Innovation, Deakin University, Geelong, VIC, Australia

**Keywords:** Artificial intelligence, Marketing, Nudge, Food, Diet

## Abstract

**Objective:**

To describe the use of artificial intelligence (AI)-enabled dark nudges by leading global food and beverage companies to influence consumer behaviour.

**Design:**

The five most recent annual reports (ranging from 2014 to 2018 or 2015 to 2019, depending on the company) and websites from twelve of the leading companies in the global food and beverage industry were reviewed to identify uses of AI and emerging technologies to influence consumer behaviour. Uses of AI and emerging technologies were categorised according to the Typology of Interventions in Proximal Physical Micro-Environments (TIPPME) framework, a tool for categorising and describing nudge-type behaviour change interventions (which has also previously been used to describe dark nudge-type approaches used by the alcohol industry).

**Setting:**

Not applicable.

**Participants:**

Twelve leading companies in the global food and beverage industry.

**Results:**

Text was extracted from fifty-seven documents from eleven companies. AI-enabled dark nudges used by food and beverage companies included those that altered products and objects’ availability (e.g. social listening to inform product development), position (e.g. decision technology and facial recognition to manipulate the position of products on menu boards), functionality (e.g. decision technology to prompt further purchases based on current selections) and presentation (e.g. augmented or virtual reality to deliver engaging and immersive marketing).

**Conclusions:**

Public health practitioners and policymakers must understand and engage with these technologies and tactics if they are to counter industry promotion of products harmful to health, particularly as investment by the industry in AI and other emerging technologies suggests their use will continue to grow.

The strategies employed by transnational food and beverage companies to promote food and beverages that are harmful to health (also known collectively as the ‘commercial determinants of health’^(1)^) are increasingly scrutinised for their role in suboptimal population diets. Food and beverage marketing is a key strategy employed by the food and beverage industry to increase the desirability and acceptability of marketed products^(1)^. Nudges are another key strategy; these seek to influence choices by making changes to the choice architecture (or the environment in which people make decisions) that ‘alter people’s behaviour in a predictable way without forbidding any options or significantly changing their economic incentives’^(2)^. Nudges are often considered in terms of how they can be used to improve diets and, in this context, include interventions such as nutrition labelling and changes to portion sizes. They do not include traditional educational efforts (for example, workshops or pamphlets), which do not change the choice architecture, nor do they include changes to prices, which change economic incentives^(3)^. While the food and beverage industry has employed some nudges to create healthy food environments^(4)^, nudges can also be used to exploit cognitive biases to promote the consumption of harmful products – these nudges have been termed ‘dark nudges’^(5)^.

Rapid technological change, such as the advent of the Internet-of-Things, associated generation of Big Data, and advances in artificial intelligence (AI), are providing opportunities for the food and beverage industry to influence customer behaviour in ways that are more intelligent, immersive and engaging than ever before. For example, McDonald’s fast food restaurant reportedly now programmes its drive-through menu boards to market products based on the time of day and weather and to display recommendations at the end of each transaction to prompt customers to order more^(6)^. Other food and beverage companies are also reportedly investing in their technological capabilities, with the Global Director of Digital Innovation at Coca-Cola describing AI as ‘the foundation for everything we do’^(7)^.

While the food and beverage industry is embracing the application of such new technologies and tactics, their use remains largely unexplored in the public health literature. Understanding and engaging with these will be essential for public health practitioners and policymakers to ensure appropriate actions are adopted to reduce the harmful influence of the food and beverage industry.

This study aims to describe the use of AI and other emerging technologies to enable dark nudges by leading global food and beverage companies to influence consumer behaviour.

For the purposes of this paper, the term ‘AI and other emerging technologies’ refer to the use of computers to autonomously or semi-autonomously complete tasks generally requiring human intelligence and to technologies such as the Internet-of-Things, robots and augmented and virtual reality. Table 1 provides definitions of the key related terms used in this paper.

**Table 1:** Key terms related to artificial intelligence (AI) and other emerging technologies

Term	Definition
Artificial intelligence	The use of computers to simulate human intelligence to autonomously or semi-autonomously complete designated tasks
Augmented reality	Technology that places customers in realistic situations that are augmented by computer-generated video, audio or sensory information
Chatbots	Automated tools that respond to customers’ enquiries
Facial recognition	Technology that identifies specific customers based on pictures or videos of their face
Geotargeting	Delivery of content based on customers’ geographic location
Image recognition	Technology that detects and identifies objects within an image
Internet-of-Things	Devices, other than computers, that can be connected to the internet and exchange information
Machine learning	The use of algorithms to find patterns in data and make decisions without explicit instruction
Natural language processing	The use of computers to process and understand spoken or written language
Recommender systems	Systems that recommend items to customers based on user and/or general behaviours and consumption patterns
Robots	Physical devices that interact with their environments through sensors and movement and complete tasks themselves
Social listening	Monitoring of what customers say online, including on social media
Virtual reality	Technology that places customers in realistic situations that are entirely generated by computers

Sources:^(43–50)^.

## Methods

We included twelve food and beverage companies in our study (Table 2). Companies were selected on the basis of being in the top five companies globally in the packaged food, soft drinks or consumer foodservice categories, using the latest available global company market shares (2019)^(8)^ at the time of selection (three companies featured in the top five companies of both the packaged food and soft drinks categories).

**Table 2:** Food and beverage companies included in review of company documents

Company (website)	Category/ies	Company headquarters	Number of countries and territories where products are sold
The Coca-Cola Co (https://www.coca-colacompany.com)	Soft drinks	Atlanta, United States	> 200
Danone Groupe (https://www.danone.com)	Packaged food, soft drinks	Paris, France	> 120
Keurig Dr Pepper Inc (https://www.keurigdrpepper.com)	Soft drinks	Burlington, United States	Not available
Kraft Heinz Co (https://www.kraftheinzcompany.com)	Packaged food	Pittsburgh, United States	Approximately 190
McDonald’s Corp (https://mcdonalds.com/)	Consumer foodservice	Chicago, United States	119
Mondelez International Inc (https://www.mondelezinternational.com)	Packaged food	Chicago, United States	> 150
Nestlé SA (https://www.nestle.com)	Packaged food, soft drinks	Vevey, Switzerland	187
PepsiCo Inc (https://www.pepsico.com)	Packaged food, soft drinks	Purchase, United States	> 200
Restaurant Brands International Inc (https://www.rbi.com)	Consumer foodservice	Toronto, Canada	> 100
Seven & I Holdings Co Ltd (https://www.7andi.com/en/)	Consumer foodservice	Tokyo, Japan	Not available
Starbucks Corp (https://www.starbucks.com)	Consumer foodservice	Seattle, United States	81
Yum! Brands Inc (https://www.yum.com/wps/portal/yumbrands/Yumbrands/)	Consumer foodservice	Louisville, United States	152

For each of the twelve included companies, we used a two-step search strategy to identify evidence of the use of AI and other emerging technologies to influence customer behaviour. First, we retrieved the five most recent annual reports available on the global websites of each of the companies in March/April 2020 (ranging from 2014 to 2018 or 2015 to 2019, depending on the company), which were read in full by the lead author (RB) to inductively identify mentions of the use of AI or other emerging technologies to directly influence customer behaviour. This included the use of broad terms such as ‘artificial intelligence’ or ‘digital technology’ or more specific terms related to specific technologies such as ‘machine learning’ or ‘image recognition’. Whether the purpose of the use of AI or other emerging technologies was to influence consumer behaviour was not always explicit; in some instances, it was inferred by the lead author (RB) based on its potential influence on selection and consumption and confirmed by the second author (DN). We did not include companies’ uses of AI and other emerging technologies to optimise food production processes as this is unlikely to have a direct impact on customer behaviour. Mentions of e-commerce, where it was not apparent that AI or other emerging technologies were employed, were also excluded.

To complement the search of annual reports, we searched the global website of each company to identify any additional company uses of AI and other emerging technologies. The websites were searched in February/March 2020 using Google and the ‘site:’ search operator. The search query was developed based on scoping searches and included both broad terms related to AI and digital technology, as well as more specific terms related to certain technologies identified during scoping, as follows:

site:URL (‘artificial intelligence’ OR AI OR ‘digital technology’ OR automate OR targeted OR ‘machine learning’ OR ‘natural language processing’ OR ‘image recognition’ OR ‘facial recognition’ OR geotarget OR bot OR ‘social listening’)

The lead author (RB) screened the first twenty pages of search results for each website for relevance (based on the title and snippet text for each item) and reviewed potentially relevant items in full.

All relevant text on the reported use of AI or other emerging technologies to influence customer behaviour from annual reports and websites was extracted into an Excel spreadsheet and categorised according to the Typology of Interventions in Proximal Physical Micro-Environments (TIPPME) framework^(9)^. The TIPPME framework is a tool for categorising and describing nudge-type behaviour change interventions that has previously been used to describe the use of dark nudge-type approaches by alcohol industry-funded corporate social responsibility organisations^(5)^. The framework categorises interventions into those related to the *placement* of products, related objects and the wider environment and those related to the *properties* of products, related objects and the wider environment^(9)^. Interventions related to *placement* are categorised according to whether they alter *availability* or *position*, while those related to *properties* are categorised by whether they alter *functionality*, *presentation*, *size* or *information* ^(9)^. While we used the TIPPME framework as a guide to categorise uses of AI or other emerging technologies, we were open to the addition of additional categories if needed.

All data extraction and categorisation were conducted by one author with expertise in public health nutrition (RB) and verified by a second author with expertise in computer engineering and AI (DN). The categorised data were reviewed, discussed and agreed upon by all authors.

Ethics approval was not required for this study as it did not involve human participants, human material or human data. All data were publicly available.

## Results

At least one mention of the use of AI or other emerging technology to influence customer behaviour was identified for eleven of the twelve leading global food and beverage companies included. Reports of AI or other emerging technology use were derived from fifty-seven documents, which included annual reports, news stories, press releases, transcripts from investor events, slides from presentations at investor events and privacy notices or policies. The level of detail reported on AI or other emerging technology use by companies varied.

When categorised to the TIPPME framework, examples of use of AI and other emerging technologies to influence customer behaviour were identified for both the *placement* and *properties* of products and related objects. Table 3 provides a summary of the TIPPME framework intervention types, their expected influence on selection and consumption and examples of AI-enabled dark nudges by the food and beverage industry, which are described in more detail in the text that follows. Supplementary Table 1 provides all extracted text and associated document details, for each TIPPME framework intervention type.

**Table 3:** Use of artificial intelligence (AI) and other emerging technologies by the food and beverage industry to enable dark nudges, identified from company documents and categorised against the TIPPME framework^(18)^

TIPPME class	TIPPME intervention type	Potential influence of intervention type on selection and consumption	Examples of AI-enabled dark nudges by the food and beverage industry
Placement	Availability *Add or remove (some or all) products or objects to increase, decrease or alter their range, variety or number*	Increasing product range may increase selection and consumption by increasing the likelihood that consumers come across a product they are willing to select or consume^(30)^ Increasing the number of discrete units of a particular product may increase selection or consumption by increasing visibility and salience^(30)^	Increasing product range based on social listening or real-times sales data Using AI-based ordering or machine learning to maintain stock levels Refining store assortments based on detailed sales data
	Position *Alter the position, proximity or accessibility of products or objects*	Increasing product proximity may increase selection and consumption by increasing visibility and salience and/or by reducing the effort required to obtain products^(30)^	Manipulating position of products on menu boards using decision technology or facial recognition Assessing the suitability of sites for restaurants using advanced technology
Properties	Functionality *Alter functionality or design of products or objects to change how they work, or guide or constrain how people use or physically interact with them*	Changes to functionality or design (such as packaging) may influence selection and consumption by changing the amount of effort required to obtain products^(31)^	Using data, analytics and decision technology to prompt further purchases online or in drive throughs based on current selections Using chatbots and voice assistants to deliver marketing to customers and facilitate ordering Delivering purchases to customers using autonomous robots
	Presentation *Alter visual, tactile, auditory or olfactory properties of products, objects or stimuli*	Changes to presentation (such as colour or brightness) may increase selection and consumption by increasing salience^(31)^	Using augmented reality and virtual reality to deliver highly engaging and immersive marketing Using automated data collection technologies and facial recognition to tailor and target marketing
	Size *Alter size or shape of products or objects*	Changes to size may influence selection and consumption due to unit bias (a heuristic that choice of one item is appropriate)^(31)^	None identified
	Information *Add, remove or change words, symbols, numbers or pictures that convey information about the product or object or its use*	Changes to the information provided (such as the inclusion of symbols) may increase selection and consumption by increasing salience, prompting use of heuristics and/or by eliciting an emotional response^(31)^	None identified

### Placement

#### Availability

Company documents show how food and beverage companies are using AI and other emerging technologies to inform seemingly more rapid, targeted and data-driven changes to their product ranges. Danone, for example, described using social listening to monitor comments on social media, through which it saw a ‘developing trend of people seeking more lactose-free choices’ and, in response, introduced a new product^(10)^. The company described how social listening allowed consumer feedback to reach companies ‘instantaneously’ and provided ‘a game changer for honing in on consumers’ needs on a local and regional level’^(10)^. In another example, The Coca-Cola Company described using real-time sales data to monitor the ‘most poured’ flavour combinations from customisable soda fountains, which it then used to ‘power the company’s innovation pipeline’, including the launch of new products^(11)^.

Company documents from a number of companies show how companies are using AI and other emerging technologies to inform product ordering and to closely monitor product levels, which can be assumed to increase the likelihood that products remain well stocked at all times. Seven & i Holdings, for example, described using AI-based ordering, which considered factors such as product-specific sales and weather-related factors, to place orders for food^(12)^, while Nestlé described using digital imaging and machine learning to monitor ice cream freezers, which allowed stores to know how much stock they had and when they would need to order more^(13)^.

PepsiCo similarly described the use of ordering algorithms to generate ‘perfect’ orders for stores, as well as investment in its ‘digital capabilities… to enable better execution of custom assortment and displays at a very granular store level’, which included the development of an app that provides ‘detailed trends at the outlet level and the store level for pack, flavor or brand’^(14)^. The combination of ‘local intuition with a much more data-driven understanding of the demographics and the consumer types in a particular store’ was described as allowing ‘much, much more precise’ store assortments, which were suggested to result in ‘double-digit types of growth’^(15)^.

#### Position

Company documents show how food and beverage companies are using AI and other emerging technologies to manipulate the position, and therefore likely selection and consumption of products. This is particularly evident in the manipulation of the visibility and salience of products displayed on menu boards. McDonald’s, for example, described using decision technology to alter its drive through menu boards based on the time of day, weather, current restaurant traffic and trending menu items^(16)^. The Coca-Cola Company similarly described a cloud-based digital signage system that allowed restaurants to update their menu boards based on real-time sales data^(17)^. Yum! Brands, meanwhile, manipulated the position of products on menu boards based on the characteristics of individual customers, with its company documents describing the use of facial recognition technology to narrow menu items shown to customers based on their estimated age, sex and apparent mood^(18)^.

Beyond the positioning of products, McDonald’s described assessing the suitability of sites for restaurants through the use of ‘advanced technology’ to analyse traffic and walking patterns, census data and other relevant data^(19)^.

### Properties

#### Functionality

Company documents show how food and beverage companies are using AI and other emerging technologies to guide consumers’ use of websites, apps and ordering kiosks or drive throughs and, in particular, cross sell products to increase the size of sales. In one example, PepsiCo described its use of data and analytics to ‘highlight relevant and contextual affinities online’ in prompting consumers to purchase additional products from its online store based on their current selections (for example, suggesting a customer purchase pita chips if they have already selected hummus), a strategy it described as having been ‘effective’^(14)^. McDonald’s similarly described using decision technology on its drive through menu boards to ‘instantly suggest and display additional items to a customer’s order based on their current selections’ and planned to integrate the same technology in other places customers make orders, such as in its self-order kiosks and app^(16)^.

Company documents also show how food and beverage companies are using AI and other emerging technologies to interact with consumers in new ways, including ways which increase the ease and convenience with which consumers can order and obtain their products and which increase the channels through which companies market to consumers. Yum! Brands, for example, described developing a bot accessible via Amazon’s voice assistant Alexa, which allowed customers to ‘use their voice to order the Colonel’s chicken’ and ‘learn about promotional offers’^(18)^. Nestlé similarly described using ‘algorithm-rich technology’ to develop ‘conversational marketing’ experiences, such as a chatbot on Facebook Messenger that delivers special offers^(20)^. PepsiCo, meanwhile, described its ‘snackbot’, a self-driving robot that delivers snacks ordered by university students via an app across a university campus, making snacking ‘ultra-convenient’^(21)^, Yum! Brands similarly described testing the use of autonomous robots to deliver pizza to customers^(18)^.

#### Presentation

Several company documents show how companies are using AI and other emerging technologies to alter presentation, particularly in the use of augmented reality and virtual reality to create highly engaging and immersive marketing campaigns. PepsiCo, for example, described the use of virtual reality in marketing that put Gatorade and Mountain Dew consumers ‘in the shoes of their favourite athletes’^(22)^, as well as the launch of ‘an artificial intelligence-powered camera’ that ‘allows users to transform everything they see into unbelievable Cheetos-inspired creations’, which can be shared on social media^(23)^. Danone similarly described launching a Snapchat lens that put evian consumers’ faces ‘in an animation of a famous evian dancing baby’^(24)^, while The Coca-Cola Company described an app with which consumers could scan products to ‘unlock’ augmented reality ‘experiences’ featuring the ‘Coca-Cola Polar Bears’^(25)^.

Company documents also show how companies are using AI and other emerging technologies (including automated data collection technologies like cookies and web beacons) to tailor the presentation of marketing and target specific marketing to specific consumers. For instance, PepsiCo described how ‘digitalizing’ its marketing and consumer insights allowed it to ‘tailor and target ads with greater precision’^(15)^, while Starbucks described using information about the location of devices consumers were using to ‘deliver more relevant advertising’, including tailored marketing offers or messages based on location, time of day and weather^(26)^. Seven & i Holdings described using facial recognition to guess customers’ age and gender and accordingly adjust the in-store advertising displayed^(27)^.

## Discussion

Our review is the first to systematically review documents from leading global food and beverage companies (which market and sell predominantly unhealthy foods and beverages (i.e. foods and beverages high in saturated fat, sodium, sugars and energy)^(28,29)^) to describe the use of AI and other emerging technologies to enable dark nudges using an established intervention typology. We found that global food and beverage companies are using a wide variety of AI and other emerging technologies to enable dark nudges that alter the availability, position, functionality and presentation of products and objects. Nudges that alter these have the potential to increase selection and consumption by exploiting a variety of cognitive biases, such as salience bias and the principle of least effort^(30,31)^. The use of AI in the identified dark nudges means that these are far more personalised and engaging than more traditional non AI-driven dark nudges, potentially driving even greater selection and consumption of unhealthy foods and non-alcoholic beverages.

Our results align with, and provide direct evidence from company documents of, some of the key features of the ‘Big Data digital food marketing system’ described in a commentary by Montgomery and colleagues, which included tracking, profiling and targeting of individual customers; geotargeting and place-based marketing; data-driven targeting by ethnicity and race; cross-device tracking and personalised advertising; in-store surveillance and point-of-purchase prompting and micro-moment messaging^(32)^. Our results also align with those of Carolan, who interviewed individuals who had overseen the use of Big Data applications within food retail settings^(33)^. The interviewees reported the use of data mining to understand customer trends (such as those based on weather or emerging food trends) and of beacon technology and media access code addresses to send alerts or coupons to customers when they were near to stores^(33)^ – we similarly found examples of product development based on social listening and of geotargeting of marketing offers or messages. The interviewees in Carolan’s study, however, also reported increasingly sophisticated purported future uses that we did not find in our study, such as the use of high-resolution cameras and software to analyse eye movement and bodily cues and send alerts to customers while they are in store^(33)^. Similarly sophisticated tactics have, however, been reported by media organisations, including the reported fitting of cameras, motion sensors and eye-tracking capabilities to frozen food aisle doors in retail settings, with information from these used to tailor the displays and promotions shown on the doors^(34)^. In a recent report, the Center for Digital Democracy documented numerous examples of the use of AI and other emerging technologies by food and beverage companies, which, similar to our results, included the use of chatbots, augmented and virtual reality, and voice assistants^(35)^.

The AI-enabled dark nudges we identified are ultimately likely to drive increased selection and consumption of targeted products, by altering the availability, position, functionality and presentation of products and objects and exploiting cognitive biases. For example, as noted in Table 3, nudges that alter the position of products may increase their selection and consumption by increasing their visibility and salience^(30)^. As the companies included in our study market and sell predominantly unhealthy foods and beverages, any increase in selection and consumption of their products is likely to reduce the quality of population diets and contribute to diet-related morbidity and mortality. While our study is not able to determine whether these AI-driven dark nudges are more effective at increasing selection and consumption than more traditional non-AI-driven dark nudges there is some evidence to suggest that this may be the case. For one, the nudges traditionally used in retail settings, such as background music, lighting, smells and product placement, are relatively blunt – for example, only one background song can be played at a time and eye-level product placement is not at eye level for all customers^(33)^. In contrast, dark nudges that employ AI and other emerging technologies can be far more personalised and engaging – for example, marketing offers sent to customers can be tailored based on customers’ location or the time of day or weather, as identified in our study, as well as large amounts of other data (such as information about individuals’ activities on websites, mobile phones and wearable devices)^(32)^. Nudges driven by such Big Data have been termed ‘hypernudges’ due to their ability to create ‘a highly personalised choice environment’ and are suggested to be ‘highly potent’^(36)^. AI and other emerging technologies that allow advertisers to generate and test multiple versions of advertisements (varying, for example, the advertisement copy, images and video) and deliver different versions of advertisements to increasingly targeted market segments are also being used and are reported to be ‘effective at driving increased sales and brand loyalty’^(35)^. Company documents included in our review also indicate that other AI-enabled dark nudges have been successful in driving sales growth. For example, PepsiCo, reported that its data-driven local store assortments had resulted in ‘double-digit types of growth’^(15)^. Further research should explore the impact of AI-enabled dark nudges on selection and consumption of intended products and total energy intake to determine their impact on population health.

The use of AI and other emerging technologies to enable dark nudges also raises several ethical concerns. The collection of personal data by technology companies through automated data collection technologies (such as cookies and web beacons) and the on-selling of these data to the food and beverage industry allows for carefully tailored and targeted marketing campaigns. This collection of personal data to market products that are harmful to health presents issues related to consent, data privacy and misuse of data^(37)^. As few consumers read or fully understand online terms and conditions, which are often lengthy and contain substantial ‘legalese’^(37)^, the extent to which consumers actually consent to the collection and use of their data to inform dark nudges is highly questionable.

There are also concerns that data can be used to target unhealthy food and beverage marketing to certain populations^(38)^, with the potential to exacerbate existing disparities in diets and health. Data related to engagement with unhealthy food marketing could also potentially be used to make inferences about long-term health risks, which could then be applied in insurance, financial services, differential pricing or other discriminatory applications^(38)^. Strengthening of data protection and privacy laws, so that they are fit-for-purpose in a highly technological and data-driven world, should be a priority of governments around the world.

In addition, regulations that specifically reduce exposure to, and the power of, marketing of unhealthy foods and beverages should be pursued by governments, as recommended by authoritative health organisations, such as the WHO^(39)^. The terms and conditions that underpin such efforts should be carefully considered and as comprehensive as possible to account for these AI-enabled dark nudges and ensure that transnational food and beverage companies do not undermine efforts to improve population diets. For example, a ban on unhealthy food marketing at end-of-aisle displays and checkouts in retail stores may be undermined by technology that determines a customer’s location within an aisle and delivers micro-targeted advertisements via mobile phone that offer discounts or rewards in real time^(32)^.

The need for this is reinforced by evidence we found in company documents (while completing this study) of large-scale investment in AI and other emerging technologies by food and beverage companies. This included McDonald’s acquisition of entire technology companies (namely Dynamic Yield, described as ‘a leader in personalization and decision logic technology’^(16)^, and Apprente, ‘an early stage leader in voice-based, conversational technology’^(40)^) and Nestlé’s description of its ‘Silicon Valley Innovation Outpost’, made up of a team of ‘commercial and technology experts’^(13)^. The outcome of this investment is likely to be increased use and increasingly sophisticated use of AI and other emerging technologies in dark nudges in future years.

Relatedly, countering the promotion of products harmful to health will require monitoring and scrutiny of the actions of not only transnational food and beverage companies but also technology companies. In addition to the role that technology companies take in the collection and sale of personal data to the food and beverage industry, technology companies are also partnering with the food and beverage industry to directly support AI-enabled dark nudges. For example, the conversational AI company Nuance Communications partnered with Domino’s Pizza to deploy a virtual assistant allowing ordering and conversations about menus, ingredients, store locations and operating hours^(41)^, while the search engine company Baidu partnered with KFC to deploy facial recognition technology to recommend menu items to customers^(18)^ (as described in our results).

Finally, although we have focused on how AI and other emerging technologies are being used to enable dark nudges, these same technologies also offer opportunities for public health. Tailoring and targeting marketing can be used to deliver health promotion messages to targeted population groups, while image recognition and machine learning technologies are beginning to be used to monitor the activities of alcohol companies^(42)^, and advanced AI technologies are increasingly being used to optimise the delivery of nutrition interventions^(43)^.

A strength of our review is the use of company documents, which provides information about the use of AI and other emerging technologies in companies’ own words. While these were from only a sample of the top global companies (by market share), these companies are likely to have the greatest resources to enable the widespread and sophisticated use of AI and other emerging technologies. While mentions of the use of AI or other emerging technologies were occasionally vague or lacking detail, authors with expertise in computer engineering and AI technologies verified that the illustrative text supported each of the included categories.

Our review was limited to documents publicly available on the sampled websites and as such is unlikely to have captured the full extent or details of the use of AI and other emerging technologies by the food and beverage industry to influence customer behaviour. Nevertheless, it provides an indication of the breadth of tactics and technologies used. Marketing literature, including grey literature, could be reviewed in future to triangulate these findings. Finally, there are a wide range of terms that may be used to describe AI and other emerging technologies. While all annual reports were reviewed in entirety, it is possible that the additional search of company websites, where we used key terms to identify relevant information, may have missed examples where alternative terms were used. However, given the likely non-technical audience for documents placed on company websites, we anticipate that most examples would have included broad terms such as ‘artificial intelligence’ or ‘digital technology’.

The use of AI and other emerging technologies to exploit cognitive biases and nudge consumers towards unhealthy foods and beverages appears widespread and sophisticated. Based on company documents, global food and beverage companies are using such technologies to enable dark nudges that alter the availability, position, functionality and presentation of products and objects and are ultimately likely to drive increased selection and consumption. Given investment by companies in AI and other emerging technologies suggests use of these technologies will only continue to grow, efforts to counter promotion of harmful products and improve population diets must be cognisant of these emerging tactics and technologies.

## Supplementary material

For supplementary material accompanying this paper visit https://doi.org/10.1017/S1368980022000490. click here to view supplementary material

## References

[1] Kickbusch I , Allen L & Franz C (2016) The commercial determinants of health. Lancet Glob Health 4, e895–e896. 27855860 10.1016/S2214-109X(16)30217-0

[2] Thaler RH & Sunstein CR (2009) Nudge: Improving Decisions about Health, Wealth and Happiness, 2nd ed. London: Penguin Books.

[3] Cadario R & Chandon P (2020) Which healthy eating nudges work best? A meta-analysis of field experiments. Marketing Sci 39, 465–486.

[4] Kraak V , Englund T , Misyak S et al. (2017) Progress evaluation for the restaurant industry assessed by a voluntary marketing-mix and choice-architecture framework that offers strategies to nudge American customers toward healthy food environments, 2006–2017. Int J Environ Res Public Health 14, 760. 28704965 10.3390/ijerph14070760 PMC5551198

[5] Petticrew M , Maani N , Pettigrew L et al. (2020) Dark nudges and sludge in big alcohol: behavioral economics, cognitive biases, and alcohol industry corporate social responsibility. Milbank Q 98, 1290–1328. 32930429 10.1111/1468-0009.12475 PMC7772646

[6] Yaffe-Bellany D (2019) Would You Like Fries With That? McDonald’s Already Knows the Answer. https://www.nytimes.com/2019/10/22/business/mcdonalds-tech-artificial-intelligence-machine-learning-fast-food.html (accessed September 2020).

[7] Marr B (2017) The Amazing Ways Coca Cola Uses Artificial Intelligence and Big Data to Drive Success. https://www.forbes.com/sites/bernardmarr/2017/09/18/the-amazing-ways-coca-cola-uses-artificial-intelligence-ai-and-big-data-to-drive-success/#2e8596178d2f (accessed October 2020).

[8] Euromonitor International (2020) Passport Global Market Information Database. https://www.portal.euromonitor.com/ (accessed February 2020).

[9] Hollands GJ , Bignardi G , Johnston M et al. (2017) The TIPPME intervention typology for changing environments to change behaviour. Nat Hum Behav 1, 1–9.

[10] Danone (2018) How Social Listening Changed What’s in your Breakfast Bowl. https://www.danone.com/stories/articles-list/social-listening-and-the-rise-of-custom-breakfast.html (accessed February 2020).

[11] The Coca-Cola Company (2019) Coca-Cola Freestyle Crowdsources Creative Drink Mixes, Rewards Fan with $10 000 Grand Prize. https://www.coca-colacompany.com/news/coca-cola-freestyle-crowdsources-drink-mixes (accessed March 2020).

[12] Seven & i Holdings (2019) Seven & i Management Report. Tokyo: Seven & i Holdings.

[13] Nestlé Innovation and Iteration. https://www.nestle.com/stories/look-at-nestle-silicon-valley-innovation-outpost (accessed March 2020).

[14] PepsiCo (2018) Edited Transcript PEP – PepsiCo Inc at Consumer Analyst Group of New York Conference. https://www.pepsico.com/docs/album/investors/2018_transcript_cagny_z36a2sufn9vkt6db.pdf (accessed March 2020).

[15] PepsiCo (2020) Edited transcript PEP - PepsiCo Inc at Consumer Analyst Group of New York Conference. https://www.pepsico.com/docs/album/investors/pep-usq_transcript_2020-02-20_wm6obg87w0cbb2ct.pdf (accessed March 2020).

[16] McDonald’s Corporation (2019) McDonald’s to Acquire Dynamic Yield, will Use Decision Technology to Increase Personalization and Improve Customer Experience. https://news.mcdonalds.com/news-releases/news-release-details/dynamic-yield-acquisition-release (accessed March 2020).

[17] The Coca-Cola Company (2018) How digital Technology and Big Data can Accelerate Coke North America’s Innovation Strategy. https://www.coca-colacompany.com/news/tech-and-big-data-accelerate-innovation-strategy (accessed March 2020).

[18] Yum! Brands (2019) 10 Ways KFC, Pizza Hut and Taco Bell are Digitally Reinventing How we Approach Food. https://www.yum.com/wps/portal/yumbrands/Yumbrands/news/company-stories/!ut/p/z0/fYxLDoIwFACv8jauWz8xuCQa44do2EE35FkKVEtfLRXE04sXcDmTyTDBMiYs9rrGoMmimTgX6yJKz4sDv_ILX0d7nh63q2SX8OUmXrATE_D6aDvz6eImZBkg3oHlo2vtrBq6IqKfDGBpHbGuwa9KuHnZ3zOYcCxg0clwenPB6F5BUBbQkBJcFPGwJRDqWsd0JgRvNK2VzZoW0NDAwwK0DlPKBuoiErmHiL_Arlp5ck!/ (accessed March 2020).

[19] McDonald’s Corporation (2019) 2018 Annual Report. Chicago: McDonald’s Corporation.

[20] Nestlé Pixel Perfect Coffee. https://www.nestle.com/stories/nespresso-digital-innovation-perfect-coffee (accessed March 2020).

[21] PepsiCo (2019) PepsiCo’s Hello Goodness Snackbot is Off to College. https://www.pepsico.com/news/press-release/pepsicos-hello-goodness-snackbot-is-off-to-college01032019 (accessed March 2020).

[22] PepsiCo (2016) 2015 Annual Report. Purchase, NY: PepsiCo.

[23] PepsiCo (2018) Cheetos Launches “Cheetos Vision” App at SXSW 2018. https://www.pepsico.com/news/press-release/cheetos-launches-cheetos-vision-app-at-sxsw-201803082018 (accessed March 2020).

[24] Danone (2018) Annual Report 2017. Paris: Danone.

[25] The Coca-Cola Company (2019) Coca-Cola Cinnamon, Augmented Reality Polar Bears and More to Deliver Holiday Magic. https://www.coca-colacompany.com/news/coca-cola-cinnamon-delivers-holiday-magic (accessed March 2020).

[26] Starbucks Coffee Company (2020) Starbucks Privacy Statement. https://www.starbucks.com/about-us/company-information/online-policies/privacy-policy (accessed March 2020).

[27] Seven & i Holdings (2019) Financial Results Presentation for the Nine Months Ended November 30, 2018. https://www.7andi.com/en/ir/file/library/ks/pdf/2019_0110kse.pdf (accessed March 2020).

[28] The George Institute for Global Health (2021) Report on the Comparative Nutritional Profile of Food and Beverage Products Marketed by the 25 Largest Global Companies in 25 Countries. Sydney: The George Institute for Global Health.

[29] The George Institute for Global Health (2020) FoodSwitch: State of the Fast Food Supply. Sydney: The George Institute for Global Health.

[30] Hollands GJ , Carter P , Anwer S et al. (2019) Altering the availability or proximity of food, alcohol, and tobacco products to change their selection and consumption. Cochrane Database Syst Rev 9, CD012573. 31482606 10.1002/14651858.CD012573.pub3 PMC6953356

[31] Ensaff H (2021) A nudge in the right direction: the role of food choice architecture in changing populations’ diets. Proc Nutr Soc 80, 195–206. 33446288 10.1017/S0029665120007983

[32] Montgomery K , Chester J , Nixon L et al. (2019) Big Data and the transformation of food and beverage marketing: undermining efforts to reduce obesity? Crit Public Health 29, 110–117.

[33] Carolan M (2018) Big data and food retail: nudging out citizens by creating dependent consumers. Geoforum 90, 142–150.

[34] Tiffany K (2019) That Freezer is Watching You. https://www.vox.com/the-goods/2019/2/7/18215629/walgreens-cooler-screens-frozen-food-eye-tracking-ads (accessed June 2021).

[35] Chester J , Montgomery KC & Kopp K (2021) Big Food, Big Tech, and the Global Childhood Obesity Pandemic. Washington: Center for Digital Democracy.

[36] Yeung K (2017) ‘Hypernudge’: big Data as a mode of regulation by design. Inf Commun Soc 20, 118–136.

[37] Mathews-Hunt K (2016) CookieConsumer: tracking online behavioural advertising in Australia. Comput Law Secur Rev 32, 55–90.

[38] Tatlow-Golden M & Garde A (2020) Digital food marketing to children: exploitation, surveillance and rights violations. Glob Food Sec 27, 100423.

[39] World Health Organization (2010) Set of Recommendations on the Marketing of Foods and Non-Alcoholic Beverages to Children. Geneva: World Health Organization.

[40] McDonald’s Corporation (2019) McDonald’s to Acquire Apprente, An Early Stage Leader in Voice Technology. https://news.mcdonalds.com/news-releases/news-release-details/McDonalds/acquire/apprente/ (accessed March 2020).

[41] Nuance Communications (2017) Domino’s Revolutionizes Digital Ordering with DRU Assist, its Virtual Assistant Powered by Nuance. https://news.nuance.com/2017-03-01-Dominos-Revolutionizes-Digital-Ordering-with-DRU-Assist-its-Virtual-Assistant-Powered-by-Nuance (accessed April 2021).

[42] Kuntsche E , Bonela AA , Caluzzi G et al. (2020) How much are we exposed to alcohol in electronic media? Development of the Alcoholic Beverage Identification Deep Learning Algorithm (ABIDLA). Drug Alcohol Depend 208, 107841. 31954949 10.1016/j.drugalcdep.2020.107841

[43] Backholer K , Lim CP , Bhatti A et al. (2020) Harnessing AI to achieve healthy and sustainable food systems. UNSCN Nutr 45, 103–106.

[44] Allen JR & West DM (2020) The Brookings Glossary of AI and Emerging Technologies. https://www.brookings.edu/blog/techtank/2020/07/13/the-brookings-glossary-of-ai-and-emerging-technologies/ (accessed April 2021).

[45] Carleton College Recommender Systems. https://www.cs.carleton.edu/cs_comps/0607/recommend/recommender/index.html (accessed April 2021).

[46] Curran T , Treiber J & Rosenblatt M (2018) Building brands through social listening. Proc Northeast Business Econ Assoc 74–77.

[47] Frankish K & Ramsey WM (2014) The Cambridge Handbook of Artificial Intelligence, 1st ed. Cambridge: Cambridge University Press.

[48] Hutson M (2017) AI Glossary: artificial intelligence, in so many words. Science 357, 19. 28684481 10.1126/science.357.6346.19

[49] Southern Oregon University (2020) How to Use Geotargeting in Marketing. https://online.sou.edu/articles/mba/how-to-use-geotargeting-in-marketing.aspx (accessed April 2021).

[50] Tricoles R (2019) What is the Internet of Things? https://research.asu.edu/what-internet-things (accessed April 2021).

