# Supplementary material for: Use of artificial intelligence to enable dark nudges by transnational food and beverage companies: analysis of company documents
Source: Public Health Nutr. 2022 Mar 2;25(5):1291–9. doi: 10.1017/S1368980022000490 (PMC9991714; doi:10.1017/S1368980022000490)
Supplement: Supplementary file 1 [file S1368980022000490sup001.docx]

Supplementary table 1: Use of AI and other emerging technologies by the food and beverage industry to enable dark nudges, identified from company documents and categorised against the TIPPME framework ^(1)^

| **TIPPME class** | **TIPPME intervention type** | **Company** | **Extracted text (source)** |
| --- | --- | --- | --- |
| Placement | Availability | The Coca-Cola Co | …Customization has been at the heart of the Coca-Cola Freestyle experience since its launch, and mixing continues to gain popularity on the platform. Fan creativity has even helped fuel some of Coke’s recent beverage innovations. By getting real-time data from each machine, the company track trending flavors and the “most poured” combinations. These insights have helped power the company’s innovation pipeline –including the 2019 launches of Sprite Lymonade and Orange Vanilla Coke…  (https://www.coca-colacompany.com/news/coca-cola-freestyle-crowdsources-drink-mixes) |
| Placement | Availability | Danone Groupe | …By listening to consumers’ comments across social channels, the company sniffed out and jumped on the developing trend of people seeking more lactose-free choices. This insight resulted in the production of Activia’s lactose-free yogurt. “Social listening puts consumers in the center of our decisions,” explains Isabel María Gonzalez, CRM and digital manager, marketing, at Danone. Besides informing the brand about ingredient preferences, social listening also helps Danone get input on packaging. A tu gusto yogurt fans asked for a different way to close the product’s pouch-style containers, and that improvement is currently in the works. Consumer requests also resulted in the four-pack—not just individual or single-cup—option. “Feedback on social media helps us stay aware of what people are saying, feeling and asking of us,” says Gonzalez. “It gives us the ability to react.” While gaining valuable insights from consumers is important, maintaining their confidence is paramount. Danone uses social listening tools and strict guidelines to help monitor brand mentions without compromising users’ privacy. “Dropping a message to a consumer when it’s relevant has actually been a welcome practice,” says Gonzalez. Social listening is a game changer for honing in on consumers’ needs on a local and regional level. Gone are the days when it would take months for consumers’ thoughts and concerns to reach the powers that be at brands. Today, they’re delivered instantaneously through tweets, snaps, Insta-stories and more…  (https://www.danone.com/stories/articles-list/social-listening-and-the-rise-of-custom-breakfast.html) |
| Placement | Availability | Danone Groupe | …To meet consumer demand with new products and flavors, we are working with data analysis tools such as Google Analytics and fast prototyping. This leads to exciting innovations such as Lemonades, launched in select cities in China. This allows us to capitalize on the flexible marketing opportunities that smaller brands provide, as well as to quickly fine-tune and deliver in-demand products to match consumer needs. Relying on these techniques, we have seen a big upturn in brands such as Ser in Argentina and Dan’Up in Mexico or France’s Les 2 Vaches, which saw double-digit growth in 2017…  (Annual report 2017: https://www.danone.com/investor-relations/publications-events/integrated-reports.html) |
| Placement | Availability | Nestlé SA | …in order to be successful, we seek out partnerships with the best and brightest entrepreneurs in Silicon Valley and beyond to help us create digital experiences and new business models. For example, we recently applied digital imaging and machine learning technology to monitor ice cream freezers, letting stores know, up to the minute, how much ice cream is in a freezer and when to order more. We are currently piloting this solution in a variety of store formats and working with a large telecommunications partner to bring the project to life on a much larger scale…  (https://www.nestle.com/stories/look-at-nestle-silicon-valley-innovation-outpost) |
| Placement | Availability | Nestlé SA | [Slide—page 18]  (https://www.nestle.com/sites/default/files/asset-library/documents/library/presentations/investors_events/investor-seminar-2019/zone-aoa.pdf) |
| Placement | Availability | PepsiCo Inc | …With the data we are collecting and that digital capabilities we are enabling for the frontline, we believe we can further improve how we plan our business, segment customers and stores and integrate in-store and online, enable on-device activation so that our consumers can find the right Frito-Lay product at the right time in the right store. For example, we are vastly improving our ordering algorithms to automatically generate the perfect order for that store based on sales histories, counter trends and inventories…  (https://www.pepsico.com/docs/album/investors/2018_transcript_cagny_z36a2sufn9vkt6db.pdf) |
| Placement | Availability | PepsiCo Inc | …Another area where we're investing to become stronger involves relevant consumer-centric innovation. For example, we're investing behind more data-driven predictive analytics to help improve cycle times and drive more incremental innovation beyond just line and flavor expansions. Past and present examples of this include premiumizing the water category with LIFEWTR and bubly, building and enhancing our Lays poppable product, expanding and growing the sports drink category with Gatorade Zero and Bolt24, introducing Game Fuel to introduce -- to address the energy needs of a targeted set of consumers and enhancing our better-for-you snacks with Simply and Off the Eaten Path, and introducing bolder and spicier flavors of existing products, such as Doritos, to address consumer preferences…  (https://www.pepsico.com/docs/album/investors/pep-usq_transcript_2020-02-20_wm6obg87w0cbb2ct.pdf) |
| Placement | Availability | PepsiCo Inc | …In the face of rapid technological innovation and accelerating change throughout our industry, we are deploying digital capabilities widely across the company. Frito-Lay North America is using Big Data to help make sure consumers can find their favorite snacks in local stores…  (2017 Annual Report: https://www.pepsico.com/investors/financial-information/annual-reports-and-proxy-information) |
| Placement | Availability | PepsiCo Inc | …At the same time, we're investing in digital capabilities for our frontline to enable better execution of custom assortment and displays at a very granular store level. We have an app called Store Facts on Demand. We can walk into a store and see immediately detailed trends at the outlet level and the store level for pack, flavor or brand, all of it, which is updated daily…  (https://www.pepsico.com/docs/album/investors/2018_transcript_cagny_z36a2sufn9vkt6db.pdf) |
| Placement | Availability | PepsiCo Inc | …So right now, our store assortments, and we have a lot of different planograms, but they're still sort of more driven by local intuition. I think when you combine local intuition with a much more data-driven understanding of the demographics and the consumer types in a particular store, you're going to get the assortment in that store much, much more precise. And we've done some work on that in Frito-Lay. And it suggests double-digit types of growth. Now that's early days, but we've learned a lot about how to get precision assortment done properly on a smaller scale. The challenge for us now is to take that technology and apply it to a much larger scale…  (https://www.pepsico.com/docs/album/investors/pep-usq_transcript_2020-02-20_wm6obg87w0cbb2ct.pdf) |
| Placement | Availability | Seven & I Holdings Co Ltd | [Slide—page 9]  (https://www.7andi.com/en/ir/file/library/ks/pdf/2019_0110kse.pdf) |
| Placement | Availability | Seven & I Holdings Co Ltd | …We have also introduced AI-based ordering for food (excluding perishables), apparel, household goods and the like. AI is attractive because it uses actual values, such as product-specific sales figures, as well as weather-related factors, to study and predict future sales trends and place orders accordingly. In addition to preventing such things as product shortages, AI-based ordering has decreased the time spent per person placing orders…  (Seven & I Management Report (June 26, 2019): https://www.7andi.com/en/ir/library/mr.html) |
| Placement | Position | The Coca-Cola Co | …The company’s focus on digitization is delivering value to retail and foodservice customers, too. …a cloud-based digital signage system lets restaurants make content updates to menu boards based on real-time purchasing data…  (https://www.coca-colacompany.com/news/tech-and-big-data-accelerate-innovation-strategy) |
| Placement | Position | McDonald’s Corp | …McDonald’s Corporation (MCD) and Dynamic Yield Ltd. today announced an agreement by which McDonald’s will acquire Dynamic Yield, a leader in personalization and decision logic technology. With this acquisition of Dynamic Yield, based in New York and Tel Aviv, McDonald’s builds on its significant technology investments for growth. McDonald’s will utilize this decision technology to provide an even more personalized customer experience by varying outdoor digital Drive Thru menu displays to show food based on time of day, weather, current restaurant traffic and trending menu items. The decision technology can also instantly suggest and display additional items to a customer’s order based on their current selections…  (https://news.mcdonalds.com/news-releases/news-release-details/dynamic-yield-acquisition-release) |
| Placement | Position | McDonald’s Corp | …The Company owns and leases real estate primarily in connection with its restaurant business. The Company identifies and develops sites that offer convenience to customers and long-term sales and profit potential to the System. To assess potential, the Company analyzes traffic and walking patterns, census data and other relevant data. The Company’s experience and access to advanced technology aid in evaluating this information…  (Form 10-K (for the fiscal year ended December 31, 2018): https://corporate.mcdonalds.com/corpmcd/investors-relations/financial-information/annual-reports.html) |
| Placement | Position | McDonald’s Corp | …The Company owns and leases real estate primarily in connection with its restaurant business. The Company identifies and develops sites that offer convenience to customers and long-term sales and profit potential to the System. To assess potential, the Company analyzes traffic and walking patterns, census data and other relevant data. The Company’s experience and access to advanced technology aid in evaluating this information…  (Form 10-K (for the fiscal year ended December 31, 2017): https://corporate.mcdonalds.com/corpmcd/investors-relations/financial-information/annual-reports.html) |
| Placement | Position | McDonald’s Corp | …The Company owns and leases real estate primarily in connection with its restaurant business. The Company identifies and develops sites that offer convenience to customers and long-term sales and profit potential to the System. To assess potential, the Company analyzes traffic and walking patterns, census data and other relevant data. The Company’s experience and access to advanced technology aid in evaluating this information…  (Form 10-K (for the fiscal year ended December 31, 2016): https://corporate.mcdonalds.com/corpmcd/investors-relations/financial-information/annual-reports.html) |
| Placement | Position | McDonald’s Corp | …The Company owns and leases real estate primarily in connection with its restaurant business. The Company identifies and develops sites that offer convenience to customers and long-term sales and profit potential to the System. To assess potential, the Company analyzes traffic and walking patterns, census data and other relevant data. The Company’s experience and access to advanced technology aid in evaluating this information…  (Form 10-K (for the fiscal year ended December 31, 2015): https://corporate.mcdonalds.com/corpmcd/investors-relations/financial-information/annual-reports.html) |
| Placement | Position | McDonald’s Corp | …The Company owns and leases real estate primarily in connection with its restaurant business. The Company identifies and develops sites that offer convenience to customers and long-term sales and profit potential to the System. To assess potential, the Company analyzes traffic and walking patterns, census data and other relevant data. The Company’s experience and access to advanced technology aid in evaluating this information…  (Form 10-K (for the fiscal year ended December 31, 2014): https://corporate.mcdonalds.com/corpmcd/investors-relations/financial-information/annual-reports.html) |
| Placement | Position | Yum! Brands Inc | …KFC is already convenient, but some KFC restaurants in China have rolled out a new facial recognition technology system that narrows a diner’s menu options based on their age, sex and whatever mood they appear to be in. If diners don’t like what’s suggested, they can see an expanded menu. Designed with Baidu, China’s largest internet search engine, the technology is meant to make it easier — and quicker — to get a meal on the go…  (https://www.yum.com/wps/portal/yumbrands/Yumbrands/news/company-stories/!ut/p/z0/fYxLDoIwFACv8jauWz8xuCQa44do2EE35FkKVEtfLRXE04sXcDmTyTDBMiYs9rrGoMmimTgX6yJKz4sDv_ILX0d7nh63q2SX8OUmXrATE_-D6aDvz6eImZBkg3oHlo2vtrBq6IqKfDGBpHbGuwa9KuHnZ3zOYcCxg0clwenPB6F5BUBbQkBJcFPGwJRDqWsd0JgRvNK2VzZoW0NDAwwK0DlPKBuoiErmHiL_Arlp5ck!/) |
| Properties | Functionality | The Coca-Cola Co | …And the next generation of the groundbreaking Coca-Cola Freestyle fountain dispenser is Bluetooth-enabled, which means Freestyle mobile app users can automatically queue up custom mixes, nutritional info and more when they step into an outlet…  (https://www.coca-colacompany.com/news/tech-and-big-data-accelerate-innovation-strategy) |
| Properties | Functionality | The Coca-Cola Co | …Back this year will be image recognition technology which will allow Australians to use their Coca-Cola products to unlock unique experiences. Consumers will be able take a snap of their Coke products, when they have come alive with colour, and win unique experiences, content and more. This year the image recognition technology will be able to recognise ‘Come Alive’ colour packs across the entire trademark - Coke, Diet Coke, Coke Zero and Coke Life, as well as recognise packs that feature in our campaign advertising…  (https://www.coca-colacompany.com/au/media-centre/media-releases/coke-to-come-alive-for-summer) |
| Properties | Functionality | The Coca-Cola Co | …And the fun doesn’t stop after the last sip. Using image recognition technology, consumers will be able to use the Coca-Cola packages – whether it be Coke, Diet Coke, Coke Zero, or Coke Life – to win unique summer experiences, unlock specialised content, and more…  (https://www.coca-colacompany.com/au/news/make-your-summer-come-alive) |
| Properties | Functionality | The Coca-Cola Co | [Slide—page 31]  (https://www.coca-colacompany.com/content/dam/journey/us/en/investors/investor-overview-q3-2019.pdf) |
| Properties | Functionality | Danone Groupe | …Blédina makes communication easy and simple with their Facebook messenger app, a 24/7 AI chatbot that uses simple language and fun emojis to ease parents’ minds and share explanations, and to help customers find answers to their baby food questions. The bot, which launches from the Blédina website, prompts users to state their question then pulls up relevant, bite-sized answers…  (https://www.danone.com/stories/articles-list/your-baby-is-up-all-night-and-so-is-bledina.html) |
| Properties | Functionality | Danone Groupe | …In 2017, we paved the way for the launch of a truly innovative personalized chatbot service to support parents in the diagnosis process of cow’s milk allergy, which enhanced our ability to support patients and ultimately deliver better care…  (2016 Integrated report: https://www.danone.com/investor-relations/publications-events/integrated-reports.html) |
| Properties | Functionality | McDonald’s Corp | …McDonald’s Corporation (MCD) and Dynamic Yield Ltd. today announced an agreement by which McDonald’s will acquire Dynamic Yield, a leader in personalization and decision logic technology. With this acquisition of Dynamic Yield, based in New York and Tel Aviv, McDonald’s builds on its significant technology investments for growth. McDonald’s will utilize this decision technology to provide an even more personalized customer experience by varying outdoor digital Drive Thru menu displays to show food based on time of day, weather, current restaurant traffic and trending menu items. The decision technology can also instantly suggest and display additional items to a customer’s order based on their current selection…  (https://news.mcdonalds.com/news-releases/news-release-details/dynamic-yield-acquisition-release) |
| Properties | Functionality | McDonald’s Corp | …McDonald’s Corporation (MCD) announced today an agreement to acquire Apprente, an early stage leader in voice-based, conversational technology. The agreement marks another bold step in advancing employee and customer facing innovations while further strengthening McDonald’s technology capabilities. The announcement comes after extensive exploration with several parties, including evaluating Apprente’s solutions in McDonald’s test restaurants. Apprente was founded in 2017 in Mountain View, California, to create voice-based platforms for complex, multilingual, multi-accent and multi-item conversational ordering. In McDonald’s restaurants, this technology is expected to allow for faster, simpler and more accurate order taking at the Drive Thru with future potential to incorporate into mobile ordering and kiosks…  (https://news.mcdonalds.com/news-releases/news-release-details/McDonalds/acquire/apprente/) |
| Properties | Functionality | McDonald’s Corp | …1. We have entered into an agreement to acquire Apprente, a Silicon Valley-based start-up. Founded in 2017, Apprente is a leader in the field of conversational voice-based technology. This will give us first-mover advantage as we continue to build a better McDonald’s. 2. Apprente’s technology will allow for faster, simpler and more accurate ordering at the Drive Thru…  (https://news.mcdonalds.com/stories/company-news-details/acquistion-of-Apprente-a-voice-based-tech-start-up/) |
| Properties | Functionality | Nestlé SA | …This is why Nespresso is on a mission to use algorithm-rich technology to create a variety of 'conversational marketing' experiences, from asking a smart speaker to trace your coffee order to telling a barista-like chatbot that you’d like a no-foam, fat-free, extra-hot latte. Launched in the US and Germany this September on Facebook Messenger, 'Nespresso Assistant' delivers everything from special offers and information on recycling, to cleaning your machine and suggesting new coffees to try. Early results are promising – 70% of users report a positive experience – and by 2020 consumers around the world will be able to order their coffee via the app without leaving Facebook Messenger. Nespresso plans to scale the platform globally over the next two years. "We think the bot will work because it’s easier than a website or proprietary app," says Leroudier. "People want simplicity, and a messaging app is up-to-date 24/7, on mobile or desktop. Conversations follow you, from the office to the home, for example. And bots are more personal, interactive and entertaining." "What type of coffee would you like to try?" Nespresso Assistant might ask you, with a cheeky smile emoji, before answering your question on machine descaling using easy-to-follow GIFs. If the bot fails to deliver, and its artificial intelligence is improving all the time – there’s no coffee jolt here. A human being is just a click away. Or just a simple voice request. Nespresso Voice Assistant is launching in France via a pilot with Google Home, and offers people similar services to the chatbot. From 2020, people will also be able to say: "OK Google, Ask Nespresso to reorder my coffee!" Your weekly coffee order just got a whole lot easier, and more fun…  (https://www.nestle.com/stories/nespresso-digital-innovation-perfect-coffee) |
| Properties | Functionality | Nestlé SA | … Nestlé Japan will soon begin using a humanoid robot to sell Nescafé machines as part of its ongoing effort to enhance brand engagement with consumers in Nescafé’s biggest market. Nescafé will use the humanoid robot Pepper to sell Nescafé Dolce Gusto and Nescafé Gold Blend Barista coffee machines in home appliance stores in Japan starting in December. Pepper is the first robot in the world that is able to read and respond to human emotion. Equipped with the latest voice and emotion recognition technology, Pepper is able to read people’s facial expressions and listen to their tone of voice to analyse how they’re feeling…  (https://www.nestle.com/media/news/nestle-humanoid-robot-nescafe-japan) |
| Properties | Functionality | Nestlé SA | …Nestlé Japan is pioneering a new consumer engagement model using the robot ‘Pepper’ – which responds to human emotions – to sell Nescafé products…  (Annual Review 2015: https://www.nestle.com/investors/publications) |
| Properties | Functionality | Nestlé SA | …In Japan we started using humanoid robots to sell Nescafé Dolce Gusto and Nescafé Gold Blend Barista coffee machines…  (Annual Report 2014: https://www.nestle.com/investors/publications) |
| Properties | Functionality | PepsiCo Inc | [Video]  (https://www.pepsico.com/news/stories/pepsico%27s-self-driving-snack-delivery-robot) |
| Properties | Functionality | PepsiCo Inc | …Meet snackbot, the outdoor, self-driving robot bringing enticing snacks and beverages from Hello Goodness, a curated portfolio of better-for-you brands from PepsiCo, to hungry college students. As part of a collaborative partnership with Bay Area-based Robby Technologies, the Hello Goodness fleet of snackbots are the first robots from a major food and beverage company in the United States to roll out, bringing great-tasting, healthier snacks and beverages direct to students, making better-for-you snacking ultra-convenient… The University of Pacific community can now order food and drinks from 9 a.m. to 5 p.m. via the snackbot App, to be delivered to more than 50 designated areas across the 175-acre campus, allowing students to enjoy their breaks between classes with a snack or beverage. The bots are ready to roll with a range of more than 20 miles on a single charge, and are equipped with camera and headlights that allow it to see and navigate carefully in full darkness or rain, as well as all-wheel drive capabilities for handling curbs and steep hills…  (https://www.pepsico.com/news/press-release/pepsicos-hello-goodness-snackbot-is-off-to-college01032019) |
| Properties | Functionality | PepsiCo Inc | …Pepsi is also putting a spin on the holiday classic "white elephant" game, asking fans to #SayItWithPepsi to win a variety of fun prizes like an ugly holiday sweater or a peppermint-themed snow tube… After communicating with a Facebook Messaging Bot, fans become eligible to win free prizes which, in true "white elephant" form, can be exchanged with their friends through Facebook messenger. At the game's end, the bot will send a unique code to all users that can be entered into Pepsi Pass, the Pepsi rewards program…  (https://www.pepsico.com/news/press-release/spread-holiday-cheer-this-season-and-sayitwithpepsi11282016) |
| Properties | Functionality | PepsiCo Inc | …Using data and analytics, we have also been able to highlight relevant and contextual affinities online. So if someone buys a Sabra hummus online, suggesting that they add Stacy's Pita Chips to that order has been effective. If you think about it, the friction of having to walk to another part of the aisle -- store and search for an affinity product is completely gone in commerce, and that's working to our advantage. Net-net, we're very pleased with our e-commerce and Frito-Lay, in particular. We doubled the business last year. We'll double it again this year…  (https://www.pepsico.com/docs/album/investors/2018_transcript_cagny_z36a2sufn9vkt6db.pdf) |
| Properties | Functionality | Seven & I Holdings Co Ltd | [Slide—page 9]  (https://www.7andi.com/en/ir/file/library/ks/pdf/2019_0110kse.pdf) |
| Properties | Functionality | Seven & I Holdings Co Ltd | …In April 2018, we started experimenting with voice-based orders in cooperation with Google’s voice AI service, “Google Assistant.”…  (Integrated Report 2018: https://www.7andi.com/en/ir/library/ar/ar_archive.html) |
| Properties | Functionality | Yum! Brands Inc | …With the help of a bot that can be accessed via Amazon’s Alexa, fans can use their voice to order the Colonel's chicken, learn about promotional offers and even hear a not-too-terrible chicken joke. If it’s a meal they’re after, the bot will connect customers with the nearest KFC and facilitate the order… The chain is also looking to robots to deliver on, well, delivery. In South Korea, the brand uses Dilly Plate, a robot waiter in a Seoul restaurant that autonomously delivers pizza to diners. In the U.S., Pizza Hut has teamed up with FedEx to test out autonomous delivery robots that take a pizza from a local restaurant to a customer’s door via the FedEx SameDay Bot  (https://www.yum.com/wps/portal/yumbrands/Yumbrands/news/company-stories/!ut/p/z0/fYxLDoIwFACv8jauWz8xuCQa44do2EE35FkKVEtfLRXE04sXcDmTyTDBMiYs9rrGoMmimTgX6yJKz4sDv_ILX0d7nh63q2SX8OUmXrATE_-D6aDvz6eImZBkg3oHlo2vtrBq6IqKfDGBpHbGuwa9KuHnZ3zOYcCxg0clwenPB6F5BUBbQkBJcFPGwJRDqWsd0JgRvNK2VzZoW0NDAwwK0DlPKBuoiErmHiL_Arlp5ck!/) |
| Properties | Presentation | The Coca-Cola Co | …Using motion tracking, sound and facial recognition, Coca-Cola Australia created a unique “happiness” experience designed to capture people’s laughter in the form of wearable, graphic art. …“We developed software that captures the data of each person’s unique smile and laughter. The computer performs an algorithm to convert the weird and wonderful sound pitches in your laugh and the movement of your smile into a 2D image. We then print that onto an environmentally sustainable T-shirt…  (https://www.coca-colacompany.com/au/news/the-art-of-happiness) |
| Properties | Presentation | The Coca-Cola Co | [Cookie policy]  (https://www.coca-colacompany.com/policies-and-practices/cookie-policy) |
| Properties | Presentation | The Coca-Cola Co | …The Coca-Cola Polar Bears also take center stage in the brand’s first large-scale Augmented Reality (AR) activation. By using the Coca-Cola mobile app to scan holiday cans and bottles, fans can explore the bears’ “arctic home” and share a little holiday magic. Different packaging options unlock different Polar Bear experiences –from a sledding scene inspired by the 2013 Coca-Cola holiday ad, to snowball fights, to a holiday light show. When two cans are scanned together, the Polar Bear family will tap out “Jingle Bells” on glass Coke bottles. Coca-Cola Digital Platforms, the company’s in-house creative agency and San Francisco-based Tactic collaborated on the AR experience…  (https://www.coca-colacompany.com/news/coca-cola-cinnamon-delivers-holiday-magic) |
| Properties | Presentation | The Coca-Cola Co | [Slide—page 31]  (https://www.coca-colacompany.com/content/dam/journey/us/en/investors/investor-overview-q3-2019.pdf) |
| Properties | Presentation | Danone Groupe | [Slide—page 77]  (https://www.danone.com/content/dam/danone-corp/danone-com/investors/en-investor-seminars/2017/day-1---17-may-2017/Investor_Seminar_-_Fit_for_innovation___growth.pdf) |
| Properties | Presentation | Danone Groupe | [Slide—page 19]  (https://www.danone.com/content/dam/danone-corp/danone-com/investors/en-investor-seminars/2018/Growth%20and%20innovation.pdf) |
| Properties | Presentation | Danone Groupe | …evian launched a partnership with Snapchat, putting a Snapcode on 500 million bottles—mainly in Europe and the U.S.—that allowed consumers to use a Snap ‘face in video’ lens to place their face in an animation of a famous evian dancing baby. These projects were a fun, interactive way to inspire consumers to connect with the brands…  (Annual report 2017: https://www.danone.com/investor-relations/publications-events/integrated-reports.html) |
| Properties | Presentation | Kraft Heinz Co | [Privacy policy]  (https://www.kraftheinzcompany.com/NewPrivacyPolicy.html) |
| Properties | Presentation | Kraft Heinz Co | [Privacy policy]  (https://www.kraftheinzcompany.com/summaryofchanges.html) |
| Properties | Presentation | McDonald’s Corp | [Privacy statement]  (https://corporate.mcdonalds.com/corpmcd/privacy.html) |
| Properties | Presentation | McDonald’s Corp | [Cookie policy]  (https://corporate.mcdonalds.com/corpmcd/cookie-policy.html) |
| Properties | Presentation | McDonald’s Corp | [Privacy policy]  (https://www.mcdonalds.com/ae/en-ae/privacy.html) |
| Properties | Presentation | McDonald’s Corp | [Privacy statement]  (https://www.mcdonalds.com/gb/en-gb/privacy-policy/full.html) |
| Properties | Presentation | McDonald’s Corp | [Privacy statement]  (https://www.mcdonalds.com/us/en-us/privacy.html) |
| Properties | Presentation | Mondelez International Inc | [Privacy policy]  (https://www.mondelezinternational.com/privacy-policy) |
| Properties | Presentation | Nestlé SA | [Privacy notice]  (https://www.nestle.com/aboutus/businessprinciples/privacy/privacy-notice) |
| Properties | Presentation | Nestlé SA | [Cookies]  (https://www.nestle.com/aboutus/businessprinciples/privacy/cookies) |
| Properties | Presentation | Nestlé SA | [How Nestlé uses cookies and other tracking technology]  (https://www.nestle.com/info/cookies) |
| Properties | Presentation | Nestlé SA | [Slide—page 31]  (https://www.nestle.com/sites/default/files/asset-library/documents/library/presentations/investors_events/investor-seminar-2019/innovation.pdf) |
| Properties | Presentation | Nestlé SA | …Our digital transformation focuses on delivering personalized messaging, services and products to consumers at scale. Powered by data and technology, we are modernizing our existing brands and business operations while developing new, digitally‑centric business models. Already 10% of all consumer contacts are personalized…  (Annual Review 2018: https://www.nestle.com/investors/publications) |
| Properties | Presentation | PepsiCo Inc | …Cheetos, one of the flagship brands from PepsiCo's Frito-Lay division, is inviting fans to see the world in a whole new light – a dangerously cheesy one – thanks to the new "Cheetos Vision" app launching today at SXSW 2018. Available on the App Store, Cheetos Vision is an artificial intelligence-powered camera that turns everything you see into Cheetos. Cheetos Vision allows users to transform everything they see into unbelievable Cheetos-inspired creations. Users can then share their signature Cheetos images and videos directly on social media. Fans can even use Cheetos Vision to transform their profile photos into dangerously cheesy Cheetos creations. "The Cheetos brand is always on the cutting edge of playful fun, so it's only natural we would leverage AI to help fans see the world through a Cheetos lens," said Rachel Ferdinando, vice president of marketing, Frito-Lay North America. "We're excited to launch Cheetos Vision alongside so many other disruptive technologies at SXSW." The Cheetos Vision launch includes ads geo-targeting attendees at SXSW 2018…  (https://www.pepsico.com/news/press-release/cheetos-launches-cheetos-vision-app-at-sxsw-201803082018) |
| Properties | Presentation | Restaurant Brands International Inc | [Privacy policy]  (https://www.rbi.com/CustomPage/Index?keyGenPage=1073752210) |
| Properties | Presentation | Restaurant Brands International Inc | [Policies]  (https://www.rbi.com/CustomPage/Index?keyGenPage=1073752211) |
| Properties | Presentation | Seven & I Holdings Co Ltd | [Slide—page 9]  (https://www.7andi.com/en/ir/file/library/ks/pdf/2019_0110kse.pdf) |
| Properties | Presentation | Starbucks Corp | [Terms of use]  (https://www.starbucks.com/rewards/terms) |
| Properties | Presentation | Starbucks Corp | [Privacy statement]  (https://www.starbucks.com/about-us/company-information/online-policies/privacy-policy) |
| Properties | Presentation | Yum! Brands Inc | [Cookies and ads policy]  (https://www.yum.com/wps/portal/yumbrands/Yumbrands/footer-pages/about-our-ads/!ut/p/z1/lZJNb4JAEIb_ih48kp0FYZcj2g8Kxu1qqbKXhgIqrbBot1r-fZfGQ2MjjZvsYZJnMm-eGSTQEokqORTrRBWySra6joXz4nLbw9Ycpmxi28AdF6b0wbfojYUWPwBceB4g8bv__m50CzxiIXNZYPoj59TfAYju-c9IIFGnRYbiIbbtHDLHwARjY2gSbLgrXZJXnGU5zVLApKXTStVqg-LmszQyqYxUlvpXKq_UAFZSqnzfq5N1_jGAel8ckrTp1XJbpM152L9pRLeLRTteI5SHpg8MpjALPeCmPQmBzyC0yDnAKHOAj8eWH3Bi0UfnBHQMiXVIcjFkoJUfivyIokruS73h-ZUCfUDBf0vRV1O87XbC07JbsV8KLa-3XZdRVFKrMd5n9Pi02mzX_f43vZdzKQ!!/) |

## References

1. Hollands GJ, Bignardi G, Johnston M *et al.* (2017) The TIPPME intervention typology for changing environments to change behaviour. *Nat Hum Behav* **1**.
